# Allostatic load in female cancer patients: a systematic review

**DOI:** 10.3389/fendo.2026.1881050

**Published:** 2026-09-09

**Authors:** Sisi Bu, Minglu Zhang, Hui Tong, Luxia Fu, Ying Ding

**Affiliations:** Department of Obstetrics and Gynecology, Nanjing Drum Tower Hospital, Nanjing, China

**Keywords:** allostatic load, allostatic overload, biomarkers, cancer, female

## Abstract

**Introduction:**

The physiological burden of chronic stress, quantified as allostatic load (AL), is implicated in cancer development and outcomes. However, a synthesis of evidence regarding its role in female-specific cancers is lacking.

**Objective:**

This systematic review aimed to synthesize existing evidence on the application of AL in female cancer patients, covering both its predictive value for cancer risk in healthy populations and its prognostic significance for outcomes and quality of life in cancer patients.

**Methods:**

Following PRISMA guidelines, we systematically searched PubMed, Web of Science, Springer Link, and Wiley Online Library from inception to June 2025. Studies investigating AL in patients with common female cancers (e.g., breast, endometrial, ovarian) were included. Study quality was assessed using the NIH Quality Assessment Tool for Observational Cohort and Cross-Sectional Studies. Data on study characteristics, AL assessment methods, associated factors, and key findings were extracted.

**Results:**

17 studies (14 on breast cancer, 2 on endometrial cancer, and 1 on ovarian cancer) were included. AL levels were influenced by sociodemographic, lifestyle, and structural factors. Higher AL was associated with an increased risk of female cancers (particularly breast cancer) incidence, poorer health-related quality of life, a higher incidence of postoperative complications (e.g., lymphedema), and increased all-cause mortality. And existing studies exhibit heterogeneity in biomarker selection and scoring thresholds.

**Conclusions:**

AL is a promising composite marker associated with the risk, prognosis, and quality of life in female cancer patients, primarily breast cancer. Current evidence is concentrated on breast cancer with limited evidence available for other types of female cancers. Future research requires standardized AL assessment and investigations across diverse female cancer populations.

**Systematic review registration:**

https://www.crd.york.ac.uk/PROSPERO/, identifier CRD420251080672.

## 1 Introduction

According to the Global Cancer Observatory (GLOBOCAN) 2022 estimates (1, 2), there were approximately 20 million new cancer cases and 9.7 million cancer-related deaths globally in 2022, and the total number of global cancer cases is projected to increase to 35.3 million by 2050, representing a 76.6% rise from the 20 million cases recorded in 2022, while cancer-related deaths are expected to reach 18.5 million, reflecting an 89.7% increase from the 9.7 million deaths reported in 2022. The burden of cancer remains a major global public health challenge.

Women have long been recognized as a priority population within healthcare systems, although historically they have been significantly underrepresented in health research. As a fundamental component of the family, their health status has a direct bearing on domestic harmony and well-being (3), while also exerting influence at both societal and economic levels (4). Women are affected by a range of female-specific malignancies, including breast cancer and gynecologic cancers (cervical, ovarian, uterine, vaginal, and vulvar). Data from 2022 indicate that breast cancer is the most commonly diagnosed cancer (2). Gynecologic cancers alone accounted for an estimated 1,473,427 new cases and 680,372 deaths worldwide in 2022 (2). It is projected that both the incidence and mortality of gynecologic cancers will continue to rise over the next two decades (2).

The diagnosis and treatment of cancer constitute a highly stressful experience for patients, often involving multiple issues that lead to emotional distress such as anxiety and fear (5). During the treatment phase, female cancer patients face stressors including diagnostic shock (6), surgical trauma (6, 7), side effects of radiotherapy and chemotherapy (8), and loss of reproductive function (9). With advancements in modern medicine and diagnostic technologies, the survival period of women with malignant tumors has extended, resulting in a large number of patients becoming “cancer survivors.” In their long-term health management, they encounter stressors such as fear of recurrence (6, 7), altered body image (10), sexual dysfunction (9, 10), and financial burden (11). Additionally, throughout the patient’s lifecycle following diagnosis and treatment, they face psychosocial pressures, including stigma (12), diminished family roles, and insufficient social support (13). These chronic stressors represent common challenges for female cancer patients.

A growing body of epidemiological and clinical research indicates that stress can influence cancer progression and metastasis, and compromise treatment efficacy by mediating glucocorticoid and/or catecholamine pathways (5). Allostatic Load (AL) is a composite index comprising biomarkers from multiple physiological systems, used to measure the cumulative wear and tear on the body caused by chronic stress (14). And Allostatic load index (ALI) was created to measure AL level (15). Research (16) indicates that compared to single biomarkers reflecting stress, a composite index of multiple biomarkers demonstrates superior efficacy in predicting health outcomes. During periods of stress, the body activates a series of neurobiological systems and releases stress hormones (such as cortisol, epinephrine, and norepinephrine) (14). These hormones are protective and adaptive in the short term, enabling the body to respond to stressors encountered in daily life (14). However, with persistent exposure to stressors, the neuroendocrine, cardiovascular, metabolic, and immune systems, as well as cellular processes, are affected. When the body fails to adequately adapt and cope, a state of physiological dysregulation—known as allostasis—emerges, which can lead to allostatic overload (14). Mechanistically, this multisystem dysregulation can promote a proinflammatory state, impair immune surveillance, and contribute to DNA damage and genomic instability, thereby creating a biological environment conducive to both cancer initiation and progression (14, 17, 18). In addition, chronic stress-related biological changes may promote tumorigenesis through epigenetic modifications that alter gene expression related to cell proliferation, apoptosis, and DNA repair (17–19). Given that AL reflects the cumulative impact of stressors over the life course, it is biologically plausible that elevated AL in healthy populations may be associated with an increased risk of developing certain cancers. Currently, AL has been widely used in research on stress-related health outcomes and has been confirmed to be associated with the onset and progression of diseases (20). Existing studies indicate that high AL is linked to an increased risk of incidence for certain cancers, such as breast cancer (21), lung cancer (22), colorectal cancer (23), and prostate cancer (24). A growing body of evidence (25–27) demonstrates that a state of high AL is associated with lower survival rates, higher risk of recurrence, more severe toxicity, and poorer quality of life in cancer patients.

Women constitute a distinct population. First, the levels and fluctuations of hormones (such as estrogen and progesterone) in females—driven by factors like the menstrual cycle, pregnancy, and menopause—significantly influence HPA axis function, immune responses, and stress perception (28–30), potentially leading to physiological stress response patterns that differ from those in males (31). Second, cancers prevalent among women, such as breast, ovarian, and cervical cancer, along with their treatments (e.g., endocrine therapy, oophorectomy), can substantially alter hormone levels and related physiological systems, which may interact with AL (28, 32). Additionally, women often occupy multiple social roles and face unique stressors—such as loss of fertility, changes in body image, and sexual dysfunction (9, 10)—which theoretically could contribute to higher AL and adverse outcomes. Existing research has applied AL to common female cancers, revealing that a high ALI is associated with an increased risk of breast cancer (21) and a higher incidence of postoperative complications following breast cancer surgery (26). However, the existing literature presents issues such as fragmentation, inconsistent findings, and significant heterogeneity in research methods. There is a lack of systematic review of studies concerning AL in female cancer patients. Consequently, the potential clinical applications of AL in female oncology—such as for risk stratification, prognostic assessment, and guidance for interventions—have not been systematically summarized or evaluated.

Therefore, this systematic review aims to comprehensively review and synthesize the existing research evidence on the application of AL across the spectrum of female cancer, encompassing both its role in cancer risk prediction among initially healthy populations and its associations with prognosis and quality of life in patients with established cancer. It seeks to deepen the understanding of the role of chronic stress-related physiological burden in the development and progression of cancer in women, thereby providing a foundation for identifying high-risk individuals, developing targeted interventions to reduce AL, and ultimately improving cancer prognosis and enhancing quality of life.

## 2 Methods

This systematic review was conducted according to the guidelines developed and recommended by the Preferred Reporting Items for Systematic Reviews and Meta-Analyses (PRISMA) group (33) and has been registered on the PROSPERO platform (CRD420251080672).

### 2.1 Search strategy

Published articles concerning allostatic load/overload were identified by searching in PubMed, Web of Science, Springer Link, and the Wiley Online Library by two investigators (Bu S and Zhang M) from inception to June 30, 2025. The search strategy was structured around three conceptual blocks combined using the Boolean operator “AND”: (1) allostatic load/overload, (2) female-specific cancer types and sex/gender terms, and (3) cancer/tumor outcomes. All terms were searched in both singular and plural forms, with the search strategy tailored for each database. The complete, unedited search strings for all four databases, along with the exact number of records retrieved per database, are provided in **Supplementary Table 1**. To minimize the risk of oversight, a supplementary search was conducted prior to finalizing the manuscript before finalizing this manuscript.

### 2.2 Study selection

We considered studies that employed allostatic load or overload as a variable in populations of women across the continuum of female-specific cancers—including studies assessing cancer risk in initially healthy populations as well as those examining outcomes in patients with confirmed breast or gynecologic cancer diagnoses. Studies that met the following criteria were included: (1) participants were female; (2) the cancer type was a common female malignancy (e.g., breast, ovarian, cervical, or endometrial cancer); (3) AL was measured and reported; (4) articles published in English. Studies were excluded if they: (1) Studies were reviews, editorials, conference abstracts, etc.; (2) Studies were not available in full text; (3) Studies did not provide details on the assessment or measurement methods for AL.

### 2.3 Quality assessment

Two investigators (Bu S and Fu L) independently assessed the quality of the included studies using specific quality assessment tools. The National Institutes of Health (NIH) Quality Assessment Tool for Observational Cohort and Cross-Sectional Studies (https://www.nhlbi.nih.gov/health-topics/study-quality-assessment-tools) was used to assess the quality of the observational studies included, and the Cochrane risk of bias assessment tool 2 (RoB 2) (34) was used to assess the quality of the randomized controlled trials.

### 2.4 Data extraction

Each article was independently reviewed by two investigators. The following data were extracted from each study: author(s), publication year, study design, cancer type, data source, sample size, types of biomarkers, number of biomarkers, method for scoring biomarkers, method for categorizing the ALI, clinical context, research topic, and main findings.

## 3 Results

### 3.1 Search results

**Figure 1:**
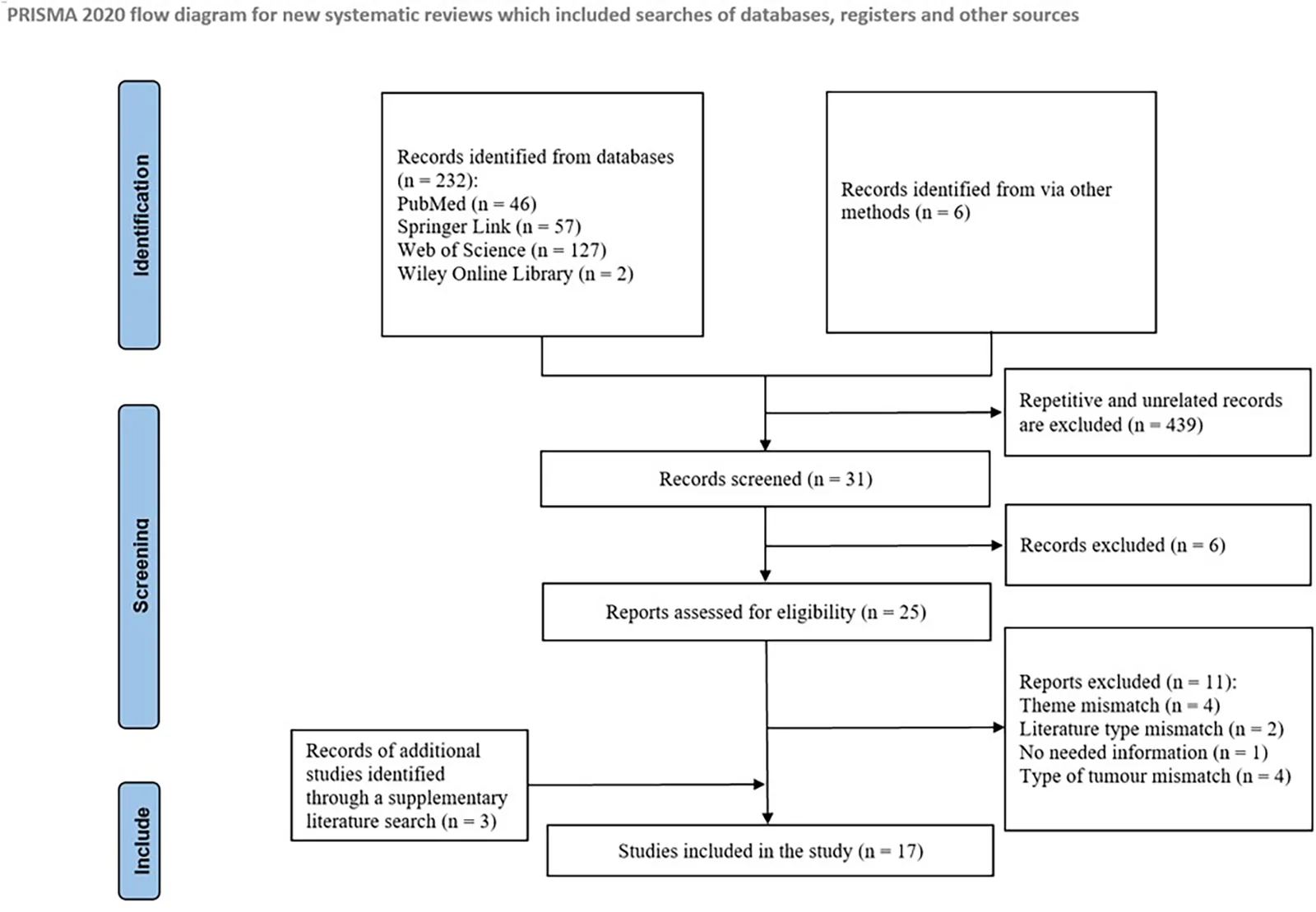
Flow chart of the identification of eligible studies.

A total of 270 studies were extracted from databases using the search strategy. After eliminating duplicate cases and unrelated articles, 31 articles remained. After reviewing titles and abstracts, 6 studies were excluded. After reading the full text, 11 articles were excluded and 14 articles (21, 26, 27, 35–45) were included. Additionally, we conducted a supplementary literature search prior to the finalization of this manuscript, which yielded 3 additional studies (46–48). Ultimately, a total of 17 studies were included in the systematic review. Flow chart is shown in **Figure 1**.

### 3.2 Study quality (risk of bias)

All included studies were observational in design. Two investigators independently assessed the quality of the included literature using the NIH Quality Assessment Tool for Observational Cohort and Cross-Sectional Studies. All included studies were rated as good or fair, and none were rated as poor. Detailed assessment results are provided in **Supplementary Table 2**.

### 3.3 Study characteristics

This systematic review included a total of 17 studies, focusing on common female cancers encompassing breast cancer (14 studies), endometrial cancer(2 studies) and ovarian cancer (1 study). The study designs were predominantly cohort studies (15 studies), supplemented by cross-sectional studies (2 studies). All studies investigated AL as a physiological indicator of cumulative biopsychosocial stress in relation to cancer risk, prognosis, quality of life, or mortality, as well as the relationship between certain factors and AL. Among the included studies, one was published in 2013, with the remaining all published within the past five years. The data sources were predominantly databases. The sample sizes across studies ranged from a minimum of 201 to a maximum of 181,455 cases. The characteristics of the included studies are presented in **Table 1**.

**Table 1:** Characteristics of included studies.

Study	Study design	Cancer type	Data source	Sample size	Biomarkers	Number of biomarkers	Method for scoring biomarkers	Method for categorizing the ALI	Clinical context	Research topic	Main findings
(41)	Cross-sectional study	Breast Cancer	National Health and Nutrition Examination Survey	4875	SBP, DBP, HR, TC, HDL, BMI, HbA1C, CRP, serum albumin	9	Categorized each selected factor as 1 or 0 based on others have used with the NHANES dataset	Using the same cutoff value (3.0) that others have used with the NHANES datasets	Determinants of AL (AL measured post-diagnosis)	Association between breast cancer and AL by race	The biological toll of breast cancer may be greater in black women than white women
(43)	Cohort study	Breast Cancer	The Women’s Circle of Health Follow-Up Study	409	SBP, DBP, WC, glucose, serum albumin, eGFR, BMI, and use of medications to control hypertension, diabetes, or hypercholesterolemia	8	Calculated using summed risk indices for each biomarker included in the computation	Using the median score as the cutoff (lower AL, 0–3 points; higher AL, 4–8 points)	Quality of life (AL measured pre-diagnosis)	Pre-diagnostic AL and HRQOL	Higher AL was associated with poorer HRQOL among Black breast cancer survivors
(44)	Cohort study	Breast Cancer	WCHFS	409	**measure1**: SBP, DBP, WC, glucose level, HDL, TC, TG, and use of medications to control hypertension, diabetes, or hypercholesterolemia **measure2**: SBP, DBP, WC, glucose level, albumin, eGFR, BMI, use of medications to control hypertension, diabetes, or hypercholesterolemia	8	Categorized each selected factor as 1 or 0 based on the specified risk threshold	Using the median of the score (3.0) as the cutoff	Cancer risk (AL measured pre-diagnosis)	Pre-diagnostic AL with breast cancer clinic pathology	Elevated pre-diagnostic AL might contribute to more unfavorable breast cancer clinicopathology
(45)	Cross-sectional study	Breast Cancer	The University of Texas M. D. Anderson Cancer Center	934	WC, BMI, SBP, DBP, HDL, LDL, TC, TG, blood glucose, HbA1C, serum albumin, eGFR, creatinine, RHR, CRP, IL-6, history of taking medication to control metabolic diseases and hypertension	17	Categorized each selected factor as 1 or 0 based on the specified risk threshold	Using the median of the score as the cutoff (median. 8 points; lower al, 0–8 points; higher al, 9–16 points).	Determinants of AL (AL measured post-diagnosis)	AL and demographics, healthy behaviors, tumor characteristics, and mitochondrial DNA	AL is influenced by selected demographics and healthy behaviors, and further is correlated with tumor characteristics and mitochondrial DNA copy number in breast cancer patients.
(40)	Cohort study	Breast Cancer	The UK Biobank	181455	SBP, DBP, CRP, HDL, LDL, TC, WHR, TG, HbA1c, creatinine, and PR	11	Categorized each selected factor as 1 or 0 based on the clinical risk threshold	0-2, ≥-	Cancer risk (AL measured pre-diagnosis)	AL and breast cancer risk	Higher AL was associated with an increased breast cancer risk in women
(27)	Cohort study	Breast Cancer	The Ohio State University Cancer Registry	2869	HR, SBP, DBP, BMI, ALP, blood glucose, albumin, creatinine, BUN, and WBC	10	Categorized each selected factor as 1 or 0 based on the cohort’s worst quartile	Using the median of the score as the cutoff	Prognosis/survival (AL measured post-diagnosis)	AL and All-Cause Mortality	AL was associated with all-cause mortality in patients with breast cancer
(35)	Cohort study	Ovarian cancer	UPMC and HCC	201	HR, SBP, DBP, WBC, ALP, albumin, BMI, glucose, creatinine, and BUN	10	Categorized each selected factor as 1 or 0 based on the cohort’s worst quartile	Using the quartiles of AL score as the cutoff (low: defined as AL in quartiles 1–3; high: defined as AL in quartile 4)	Prognosis/survival (AL measured post-diagnosis)	AL and overall survival	High AL was associated with a significant increase in mortality
(36)	Cohort study	Breast Cancer	A National Cancer Institute-designated Comprehensive Cancer Center	2772	HR, SBP, DBP, BMI, ALP, glucose, albumin, WBC, BUN, creatinine	10	Categorized each selected factor as 1 or 0 based on the cohort’s worst quartile	Using the median (interquartile range) [2.0 (3.0)] of the score as the cutoff	Determinants of AL and prognosis/survival (AL measured post-diagnosis)	Racialized economic segregation and AL	Racialized economic segregation is associated with high AL and a greater risk of all-cause mortality in patients with breast cancer.
(37)	Cohort study	Breast Cancer	The Ohio State Cancer registry	4459	Not described specifically	Not described specifically	Not described specifically	Using the median of the score (2.0) as the cutoff	Treatment complications	AL and postoperative complications	AL was associated with higher odds of postoperative complications
(38)	Cohort study	Breast Cancer	The Ohio State University Comprehensive Cancer Center	4089	HR, SBP, DBP, BMI, ALP, blood glucose, albumin, WBC, creatinine, and BUN	10	Categorized each selected factor as 1 or 0 based on the cohort’s worst quartile	Using the cohort’s median score (2.0) as the cutoff	Determinants of AL and prognosis/survival (AL measured post-diagnosis)	Neighborhood Opportunity, AL, and All-Cause Mortality	Lower neighborhood opportunity was associated with higher AL and greater risk of all-cause mortality among patients with breast cancer
(21)	Cohort study	Breast Cancer	The Women’s Health Initiative (WHI)	27393	PR, SBP, DBP, BMI, WC, CRP, glucose, and TC	8	Categorized each selected factor as 1 or 0 based on other study	Low, medium, and high (based on the tertile distribution of their scores)	Cancer risk (AL measured pre-diagnosis)	AL and risk of invasive breast cancer	Higher AL was significantly associated with an increased risk of breast cancer in postmenopausal women
(39)	Cohort study	Breast Cancer	The University of Virginia Comprehensive Cancer Center in the last decade (2014-2024)	3069	HR, SBP, DBP, BMI, TG, HDL-C, LDL-C, TC, ALP, fasting glucose, albumin, creatinine levels, eGFR, BUN, WBC, and medication history for diabetes, cardiovascular disease, chronic kidney disease, and hypertension	16	Each biomarker was dichotomized based on established clinical risk thresholds, assigning a score of 1 or 0	Based on the distribution of scores within the study population, low AL (score ≤3) and high AL (score >3)	Determinants of AL and prognosis/survival (AL measured post-diagnosis)	AL and Racial and Rural Disparities in Breast Cancer Survival	High AL was independently associated with worse overall survival
(26)	Cohort study	Breast Cancer	The Ohio State University Cancer Registry	3609	HR, SBP, DBP, BMI, ALP, glucose, albumin, creatinine, BUN, and WBC	10	Categorized each selected factor as 1 or 0 based on the cohort’s worst quartile	Using the median of the score (2.0) as the cutoff and based on the categorized into quartiles (0–1, 2–3, 3–4, 5 +).	Treatment complications (AL measured post-diagnosis)	AL and lymphedema	High AL at diagnosis was associated with higher odds of developing lymphedema
(42)	Cohort study	Breast Cancer	Pathways Study	2553	SBP, DBP, TC, HDL, TG, PR, BMI, WHR, WC, fasting glucose, CRP, WBC, and asthma diagnosis	13	Categorized each selected factor as 1 or 0 based on the cohort’s worst quartile	Using the cohort’s median score (3.0) as the cutoff	Treatment complications (AL measured post-diagnosis)	Neighborhood stressors and AL	Low nSES, high traffic, high crime, high household crowding, high proportion of fast-food restaurants or unhealthy retail food outlets, and less green space were associated with high AL
(49)		Endometrial Cancer	MD 164 Anderson Cancer Center	398	SBP, DBP, HDL, TC, TG, WC, BMI, RHR, HbA1c, albumin, CRP, IL-6, eGFR, creatinine, fasting glucose, and use of medications to control hypertension, diabetes, or hypercholesterolemia	15	Used the distribution-based approach	Used the distribution-based approach(0–3, 4–15)	Prognosis/survival (AL measured post-diagnosis)	AL and survival outcomes	For women with low-grade tumors, higher AL was associated with poorer overall survival. For high-grade tumors, intermediate AL were associated with shortest overall survival
(47)	Cohort study	Breast Cancer	The UK Biobank	159240	SBP, DBP, CRP, LDL, HDL, fasting glucose, BMI, TC, TG, WBC, albumin, and hemoglobin	12	Categorized each selected factor as 1 or 0 based on the cohort’s worst quartile	Using the cohort’s median score (3.0) as the cutoff	Cancer risk (AL measured pre-diagnosis)	AL and the risk of breast carcinoma	AL is an independent risk factor for invasive breast cancer, it shows no association with breast carcinoma in situ
(48)	Cohort study	Endometrial Cancer	A single academic center within a large healthcare delivery system in North Carolina	2174	SBP, DBP, PR, BMI, ALP, blood glucose, creatinine, BUN, WBC, and serum albumin	10	Categorized each selected factor as 1 or 0 based on the cohort’s worst quartile	Using the cohort’s median score as the cutoff	Prognosis/survival (AL measured post-diagnosis)	AL and overall survival	Elevated AL was associated with worse overall survival

SBP, Systolic Blood Pressure; DBP, Diastolic Blood Pressure; HR, Heart Rate; RHR, Resting Heart Rate; PR, Pulse Rate; BMI, Body Mass Index; WC, Waist Circumference; WHR, Waist-to-Hip Ratio; TC, Total Cholesterol; TG, Triglycerides; HDL-C, High-Density Lipoprotein Cholesterol; LDL-C, Low-Density Lipoprotein Cholesterol; HbA1c, Glycated Hemoglobin A1c; eGFR, Estimated Glomerular Filtration Rate; BUN, Blood Urea Nitrogen; CRP, C-Reactive Protein; WBC, White Blood Cell Count; IL-6, Interleukin-6; ALP, Alkaline Phosphatase; AL, Allostatic Load; ALI, Allostatic Load Index; WCHFS, The Women’s Circle of Health Follow-Up Study; UPMC, The University of Pitts-burgh Medical Center; HCC, Hillman Comprehensive Cancer Center; HRQOL, health-related quality of life; nSES, neighborhood socioeconomic status.

### 3.4 AL assessment methods

The number of biomarkers used to assess AL varied across studies (8–17 items), encompassing the cardiovascular system, metabolic system, immune system, liver and kidney function, as well as disease and medication history. A total of 23 biomarkers were involved, including: Systolic Blood Pressure (SBP), Diastolic Blood Pressure (DBP), Heart Rate (HR), Resting Heart Rate (RHR), Pulse Rate (PR), Body Mass Index (BMI), Waist Circumference (WC), Waist-to-Hip Ratio (WHR), Total Cholesterol (TC), Triglycerides (TG), High-Density Lipoprotein Cholesterol (HDL-C), Low-Density Lipoprotein Cholesterol (LDL-C), Glucose, Glycated Hemoglobin A1c (HbA1c), Hemoglobin, Creatinine, Estimated Glomerular Filtration Rate (eGFR), Blood Urea Nitrogen (BUN), Albumin, C-Reactive Protein (CRP), White Blood Cell Count (WBC), Interleukin-6 (IL-6), Alkaline Phosphatase (ALP), disease (asthma diagnosis) and medication history. The number of biomarkers used varied across studies (ranging from 8 to 17). Systolic and diastolic blood pressure were included in all studies. Other frequently used biomarkers (appearing in ≥10 studies) included HR/PR, glucose, creatinine, TC, serum albumin, HDL, and WBC. All studies employed a dichotomous scoring method to assign values to each biomarker (1 point: indicating the biomarker was in a “high-risk” state; 0 points: indicating it was in a “normal or low-risk” state). The methods for determining high-risk thresholds primarily followed three approaches: (1) Based on clinical guidelines or established risk thresholds (39, 40, 45); (2) Based on the distribution of the study cohort (the most common method): using the worst quartile as the high-risk threshold (26, 27, 37, 42, 46–48), with a few studies using tertiles or specific percentiles; (3) Referencing thresholds commonly used in previous similar studies or databases (41). The scores (0 or 1) of all selected biomarkers were summed to obtain the ALI. Based on the total score, studies categorized participants into different ALI groups. Commonly, the median or a clinically empirical value (e.g., 3) was used as the cut-off point to dichotomize the ALI into low-risk and high-risk groups. The study (21) by Wang et al. classified the ALI into low, medium, and high groups according to tertiles of the total score. The study (35) by Borho et al. used the worst quartile of the total score for categorization.

### 3.5 AL associated factors

All studies included in this systematic review examined factors associated with AL, which encompassed a wide range of aspects including: age, education, race/ethnicity, marital status, place of residence, income, insurance type, retirement status, smoking history, alcohol consumption history, medical history, physical activity, diet, sleep patterns, comorbidities, age at menarche, age at first birth, oral contraceptive use, hormone replacement therapy, mammography screening, parity, and duration of breastfeeding. Higher AL was consistently associated with older age, Black race, lower socioeconomic status (including lower education, income, public insurance, unemployment, and marital dissolution), and unhealthy lifestyle behaviors such as smoking, physical inactivity, and poor sleep quality (27, 40, 45, 48). Among reproductive factors, earlier age at menarche and younger age at first live birth were linked to elevated AL, while nulliparity was more common among women who developed breast carcinoma *in situ* compared with those who did not (40, 47). Beyond individual-level factors, neighborhood conditions also played a role. Higher AL was associated with lower neighborhood socioeconomic status, higher crime rates, greater household crowding, higher density of fast-food restaurants, less green space, lower neighborhood opportunity, racialized economic segregation, and rural residence (37–39, 42). See **Table 2** for details.

**Table 2:** Factors associated with allostatic load across included studies.

Study	Demographic & SES	Lifestyle & reproductive	Neighborhood	Tumor characteristics
(41)	Black (+)	NR	NR	NR
(43)	US-born (0), marital status (0)	BMI (+ for inflammatory AL)	NR	NR
(44)	Age (0), marital status (0)	BMI (0)	NR	Poorly differentiated (+), larger size (+), ER− (suggestive)
(45)	Black (+), Hispanic (+), divorced/widowed/separated (+), lower education (+), older age (+)	Smoking (+), physical inactivity (+), alcohol (0)	NR	Poorly differentiated (+), ER− (+ in Black)
(40)	Age (+), Black (+), lower income (+), public insurance (+), retired (+), non-married (+), lower education (+)	Smoking (+), physical inactivity (+), alcohol (−), early menarche (+), early first birth (+), HRT (+), nulliparity (+)	NR	NR
(27)	Black (+), single (+), widowed/separated/divorced (+), Medicaid/Medicare (+), older age (+)	HR−/HER2− (+)	NR	Stage III (+)
(21)	Black (+), lower education (+), lower income (+), public insurance (+), non-Hispanic (+), older age (+)	Higher parity (+), younger menarche (+), no HRT (+), breastfeeding (+)	Region (South) (+)	NR
(35)	Race (0), age (0), marital status (0)	NR	NR	Histology (0), stage (0), debulking (0), platinum sensitivity (0)
(37)	NR	NR	Lower opportunity (+), low education (+), poor housing (+)	NR
(38)	Black (+), Medicaid (+), single/widowed/divorced (+), smoking (+)	NR	ICE segregation (+)	TNBC (+), stage III (+), high CCI (+)
(42)	Lower nSES (+), NH-Black (highest AL)	Physical inactivity (+)	Low nSES (+), traffic (+), crime (+), crowding (+), fast-food (+), less green space (+)	NR
(39)	Black (+), rural (+), lower education (+), lower income (+), public insurance (+), unemployed/retired (+), non-married (+), older age (+)	Smoking (+), never drinking (+), postmenopausal (+)	Rurality (+); ADI (0)	ER+ (+); TNBC (0); stage (0)
(26)	Black (+), Medicaid (+), younger age (+)	NR	NR	Stage III (+), HR−/HER2− (+), ALND (+)
(47)	Higher SES (college, employed, higher income) associated with BCIS (screening bias)	Physical inactivity (+ for BCIS), nulliparity (+ for BCIS)	NR	NR
(48)	Black (+), non-married (+), public insurance (+), higher BMI (+)	NR	NR	Carcinosarcoma/clear cell/mixed (+); serous (−)
(49)	Black (highest AL 4.81), SVI (+)	NR	SVI (+)	High-grade (+ in Black); AL-survival varies by grade/race

“+” indicates positive association (higher AL associated with the factor); “−” indicates inverse association; “0” indicates null/no association; “NR” indicates not reported. AL, allostatic load; SES, socioeconomic status; nSES, neighborhood socioeconomic status; ICE, Index of Concentration at the Extremes; ADI, Area Deprivation Index; SVI, Social Vulnerability Index; CCI, Charlson Comorbidity Index; HRT, hormone replacement therapy; ER, estrogen receptor; TNBC, triple-negative breast cancer; BCIS, breast carcinoma *in situ*; IDC, invasive breast cancer; ALND, axillary lymph node dissection; NH-Black, non-Hispanic Black.

### 3.6 Relationship between AL and disease outcomes

Several cohort studies (21, 40) have shown that a higher ALI is associated with an increased risk of breast cancer incidence, particularly among postmenopausal women. A recent large-scale UK Biobank study further demonstrated that ALI is independently associated with an increased risk of invasive breast cancer but not with breast carcinoma *in situ* (47), suggesting that chronic physiological stress may play a more prominent role in promoting invasive progression rather than initiating *in situ* lesions. Elevated ALI is significantly correlated with poorer health-related quality of life (HRQOL) (43), especially affecting Black breast cancer survivors. High ALI is also associated with a greater risk of postoperative complications (36), lymphedema incidence (26), and all-cause mortality (27, 37). Women with ER-positive breast cancer had a higher mean AL score than those with ER-negative disease, and deceased patients during follow-up exhibited significantly higher mean AL scores than survivors (21). Furthermore, one study (45) found that AL is associated with tumor characteristics and mitochondrial DNA copy number, suggesting its potential influence on tumor biology. In gynecologic cancers, a study on ovarian cancer (35) indicated that a high ALI is significantly associated with shorter overall survival. This association has been further extended to endometrial cancer, where higher ALI was also linked to worse overall survival (48), although the relationship may vary by tumor grade and race/ethnicity (49).

## 4 Discussion

This systematic review synthesizes evidence from 17 studies investigating the relationship between AL and common female cancers, primarily breast cancer. The findings demonstrate that AL, as a composite measure reflecting the cumulative burden of chronic physiological stress, is associated with the risk of developing these cancers (notably breast cancer), their clinicopathological features, patient prognosis, and quality of life. Furthermore, AL levels are influenced by a range of factors including general characteristics, lifestyle, and social and environmental structural determinants.

The studies included in this systematic review explored the relationships between various factors and AL, encompassing patient demographics, lifestyle, disease-related factors, socioeconomic status, racialized economic segregation, and community-level stressors. Although some findings showed inconsistencies, which may be attributed to differences in sample characteristics, AL calculation methods, or the selection of control variables, the majority of the evidence indicates that older age, Black race, unmarried/unpartnered status, lack of health insurance, smoking history, and alcohol consumption are associated with higher AL levels, whereas higher income, higher education level, and greater physical activity are associated with lower AL levels in female cancer patients. This finding aligns with results from studies involving other populations, such as elderly cancer patients and male cohorts (50–52), further supporting the generalizability of AL as a physiological marker of the social gradient in health. We also identified associations between AL and social determinants of health, where lower socioeconomic status, higher racialized economic segregation, and greater community-level stressors were associated with higher AL levels.

Delving into the implications of these associations within the cancer population is particularly important. These factors do not exist in isolation; they collectively shape AL through cumulative and interactive effects. For example, lower socioeconomic status may restrict access to healthy food and healthcare resources while increasing exposure to chronic stress, thereby jointly elevating AL. Under the significant stressor of a cancer diagnosis, this pre-existing high AL state may be further exacerbated, forming a vicious cycle of “stress-physiological wear-poor prognosis.” The AL in cancer patients reflects not merely their state around the time of diagnosis but rather the physiological culmination of lifelong social, economic, and environmental pressures. This suggests that intervention strategies for cancer need extend beyond purely clinical treatment to integrate public health and social policies, aiming to alleviate the structural stressors that contribute to high AL at their root.

It is particularly noteworthy that in the context of cancer, the relevance of certain factors may be intensified or acquire new clinical significance. AL here may serve as a pathway through which “exposures” translate into “biological consequences.” For instance, racial disparities manifested in AL are not only linked to general health risks but are also intricately intertwined with the more aggressive tumor biology and higher mortality rates observed in breast cancer (53). Specifically, the higher prevalence of triple-negative breast cancer among Black women and its poorer outcomes (54). AL is associated with both Black race and more aggressive tumor biology. The same relationship applies to age of menarche and breast cancer, where earlier menarche is associated with elevated AL and, in turn, with more aggressive molecular tumor features and worse prognosis (55). This pattern extends to endometrial cancer, where Black patients exhibited the higher AL scores and the higher mortality risk, particularly among those with high AL (48), and the relationship between AL and survival further varied by tumor grade and race/ethnicity (49). These observations suggest that AL may be one of the biological pathways through which certain factors contribute to health disparities in cancer. Similarly, the impact of lifestyle factors (e.g., smoking, physical inactivity) on AL may correlate with treatment tolerance, risk of complications, and recurrence rates in cancer patients, potentially positioning AL as a mediating variable connecting behavioral risks to cancer outcomes (56, 57).

In addition, given the unique characteristics of the female cancer population, some studies incorporated specific factors such as age at menarche, age at first birth, oral contraceptive use, hormone replacement therapy, mammography screening, parity, and duration of breastfeeding. The relationships between these factors and AL warrant further investigation, which could offer valuable insights for future research focusing on AL in female cancer patients.

This systematic review suggests that higher AL levels are associated with an increased risk of female cancers(particularly breast cancer), shorter overall survival after diagnosis, a higher incidence of postoperative complications such as lymphedema, and reduced health-related quality of life. These findings are consistent with existing research (18, 22, 24, 58) on AL in other populations. The evidence supports the predictive value of AL across multiple stages of the cancer natural history, from pre-diagnosis to post-diagnosis. The underlying biological mechanism may be that the dysregulation across the cardiovascular, metabolic, inflammatory/immune, and neuroendocrine systems encompassed by AL collectively creates a “soil” that fosters tumor initiation and progression (59). For instance, chronic inflammation (elevated CRP) and insulin resistance (abnormal blood glucose or HbA1c) are established facilitators of carcinogenesis (60, 61), while sustained sympathetic nervous activation (elevated blood pressure and heart rate) may accelerate disease progression by affecting the tumor microenvironment and immune surveillance (62). Consequently, AL might transcends individual biomarkers, offering a window into an individual’s overall state of “physiological wear and tear.” This may can provide a novel perspective for identifying individuals at high risk for cancer and patients with a poor prognosis.

Of particular note, one study included in our review (45) further suggested an association between AL and tumor characteristics as well as mitochondrial DNA copy number. This finding suggests that the systemic physiological dysregulation represented by AL may not merely create a passive, cancer-promoting “soil,” but could actively intrude into and reshape the intrinsic biology of the tumor. Supporting this notion, emerging evidence from the UK Biobank cohort demonstrated that AL was independently associated with invasive breast cancer but not with breast carcinoma *in situ* (47), implying that a compromised systemic milieu may be particularly critical for enabling localized lesions to acquire an invasive phenotype—a process that likely involves fundamental alterations in tumor biology rather than mere promotion of initial malignant transformation. These observations raise the possibility that AL may represent more than a correlative marker of risk and prognosis, and might be involved in the biological processes underlying tumor progression. However, given the observational nature of the included studies, this hypothesis remains tentative and requires confirmation through mechanistic and experimental research.

Although partial results from the included studies show consistency, this systematic review also reveals heterogeneity in current methodological approaches to AL research, primarily manifested in the following three aspects. First, the selection of biomarkers. There are variations across studies in both the number (8–17) and specific types of biomarkers included. Although multiple physiological systems are typically encompassed, a universally accepted “gold standard” combination is lacking. Second, the definition of risk thresholds for biomarkers. Most studies rely on internal percentile distributions of the study cohort (e.g., the worst quartile) to define high risk. While this can reflect relative risk within the population, it compromises absolute comparability across different studies or populations. Third, the classification criteria for the ALI are inconsistent. Methods range from dichotomization (low/high) to multi-category approaches (e.g., quartile-based grouping), which affects the precise characterization of risk gradient effects. This heterogeneity, while stemming from differences in research objectives and data, limits the direct comparison and pooled analysis of study findings and may hinder the standardized application of AL in clinical practice. This is a common issue present in current AL research across different populations (15).

Within the female cancer patient population, biomarkers currently used to construct AL in existing studies are primarily based on HR/PR, blood glucose, creatinine, TC, serum albumin, HDL-C, and WBC, and are supplemented with additional indicators based on available resources and specific research questions. However, it should be noted that the appropriateness of using certain indicators warrants careful evaluation given the unique context of cancer diagnosis and treatment. For example, WBC counts are often actively managed and normalized during cancer radiotherapy and chemotherapy. Future research could utilize the Delphi method or large-scale validation cohort studies to establish one or more standardized ALI calculation protocols for specific populations or diseases, incorporating a core set of biomarkers with unified clinical thresholds.

The evidence synthesized in this systematic review is highly concentrated on breast cancer (14 studies), with 2 studies examining endometrial cancer (48, 49), and 1 study involving ovarian cancer (35). No studies investigating AL in cervical cancer or other common female malignancies were identified. The current predominance of breast cancer research is likely attributed to its epidemiological prominence as the most common female malignancy (63), its well-established hormonal associations (64), and the availability of large, well-established cohorts dedicated to women’s health. While this has contributed to a relatively well-established evidence base for AL in breast cancer, it also limits the generalizability of the findings to other female populations, which differ fundamentally from breast cancer in their etiology, pathogenesis, treatment modalities, and prognosis. The preliminary evidence on endometrial cancer is suggestive but derived from single-center retrospective studies with modest sample sizes (48, 49); therefore, these findings should be interpreted cautiously and require replication in larger, more diverse cohorts. Therefore, future research should consider investigating AL in female cancers with significant disease burden and strong links to socioeconomic factors in their causation or prognosis, such as endometrial, ovarian and cervical cancers. This would help build a more complete and broadly relevant scientific understanding, ultimately unlocking AL’s potential for improving health outcomes for all female cancer patients.

And, the substantial clinical heterogeneity across the included studies—spanning cancer risk, prognosis/survival, treatment complications, quality of life, and determinants of AL—limits the feasibility of direct comparisons or meta-analysis. We therefore present this review as a scoping synthesis of the current landscape, rather than a confirmatory meta-analysis. Future research should prioritize endpoint-specific investigations to enable more definitive conclusions. In addition, the studies included in this review are predominantly observational (especially cohort studies). Therefore, while they reveal strong associations, they cannot fully establish causality. More prospective and mechanistic studies are needed in the future to elucidate the specific pathways through which AL influences tumor biology.

This study explored the current application of AL in female cancer populations within existing research, and the findings hold significant translational value. At the clinical level, it is conceivable that ALI might serve as a simple auxiliary tool in oncology for risk stratification of patients at initial diagnosis, helping to identify those with heightened physiological wear and tear who may face a more complex disease course and poorer outcomes, thereby triggering closer monitoring or supportive therapies. At the public health level, AL could hypothetically function as a quantifiable indicator for assessing community health risks and the impact of environmental stressors, guiding the allocation of resources toward communities with a high ALI burden and facilitating targeted cancer prevention and health promotion programs. This study suggests that future research could develop and validate standardized AL assessment tools to facilitate their integration into clinical decision support systems; conduct multi-center, cross-cancer-type cohort studies to clarify the role of ALI across different tumor biological contexts; design intervention studies aimed at reducing ALI to evaluate whether it can improve cancer prevention and patient outcomes; and deeply explore the molecular mechanisms through which AL influences tumor biology, thereby providing novel insights for developing new adjuvant therapeutic strategies.

## 5 Conclusion

This systematic review synthesizes current evidence on the relationship between AL and female cancers (primarily breast cancer). The results indicate that AL levels are influenced by general characteristics, lifestyle, and social-structural and environmental factors. Higher AL levels are associated with an increased risk of developing common female cancers (particularly breast cancer), shorter overall survival, a higher incidence of postoperative complications such as lymphedema, and reduced health-related quality of life. Existing studies exhibit heterogeneity in biomarker selection and scoring thresholds. The current evidence is highly concentrated on breast cancer. Future multi-center, cross-cancer-type cohort studies should be conducted to elucidate the role of AL across different tumor biological contexts in women.

## Data Availability

The original contributions presented in the study are included in the article/**Supplementary Material**. Further inquiries can be directed to the corresponding authors.

## References

[1] Bray F Laversanne M Sung H Ferlay J Siegel RL Soerjomataram I . Global cancer statistics 2022: GLOBOCAN estimates of incidence and mortality worldwide for 36 cancers in 185 countries. CA: A Cancer J For Clin. (2024) 74:229–63. doi: 10.3322/caac.21834 38572751

[2] Bizuayehu HM Ahmed KY Kibret GD Dadi AF Belachew SA Bagade T . Global disparities of cancer and its projected burden in 2050. JAMA Netw Open. (2024) 7:e2443198. doi: 10.1001/jamanetworkopen.2024.43198 39499513 PMC11539015

[3] Chu L Zhang Q . Impact of the input of women's working hours on household non-economic welfare. BMC Public Health. (2024) 24:3523. doi: 10.1186/s12889-024-21063-x 39695545 PMC11658300

[4] Eick SM Eatman JA Chandler M Brooks NR . Reproductive and social policies, sociopolitical stress, and implications for maternal and child health equity. Curr Environ Health Rep. (2024) 11:279–87. doi: 10.1007/s40572-024-00443-w 38639910 PMC11531301

[5] Huang Y Zhan Y Zhan Y . Psychological stress on cancer progression and immunosenescence. Semin Cancer Biol. (2025) 113:85–99. doi: 10.1016/j.semcancer.2025.05.007 40348001

[6] Lakhiani A Cummins C Kumar S Long J Arora V Balega J . Analysis of anxiety, depression and fear of progression at 12 months post-cytoreductive surgery in the SOCQER-2 (Surgery in Ovarian Cancer-Quality of Life Evaluation Research) prospective, international, multicentre study. Cancers. (2023) 16:75. doi: 10.3390/cancers16010075 38201503 PMC10778036

[7] He J Zhang Y . Analysis of risk factors for negative emotions in perioperative period of ovarian cancer patients and their impact on prognosis. Gland Surg. (2023) 12:492–507. doi: 10.21037/gs-23-94 37200931 PMC10186174

[8] Gaumond SI Beraja GE Kamholtz I Ferrari LM Mahmoud RH Jimenez JJ . Chemotherapy-induced alopecia in ovarian cancer: incidence, mechanisms, and impact across treatment regimens. Cancers. (2025) 17:411. doi: 10.3390/cancers17030411 39941780 PMC11816305

[9] Cosyns S Dony N Polyzos N Buyl R Tournaye H Schotte C . Impact of diagnosis and surgical treatment of early stage borderline ovarian tumours on distress, anxiety, and psychosexual health. J Psychosomatic Obstetrics Gynecology. (2024) 45:2404010. doi: 10.1080/0167482X.2024.2404010 39301872

[10] Boding SA Russell H Knoetze R Wilson V Stafford L . 'Sometimes I can't look in the mirror': recognising the importance of the sociocultural context in patient experiences of sexuality, relationships and body image after ovarian cancer. Eur J Cancer Care. (2022) 31:e13645. doi: 10.1111/ecc.13645 35790894 PMC9787468

[11] Spagnoletti B Bennett LR Keenan C Shetty SS Manderson L McPake B . What factors shape quality of life for women affected by gynaecological cancer in South, South East and East Asian countries? A critical review [Journal Article; Review. Reprod Health. (2022) 19:70. doi: 10.1186/s12978-022-01369-y 35305676 PMC8934499

[12] Bae HS Temkin SM . Cervical cancer stigma - a silent barrier to the elimination of cervical cancer. Cancer. (2025) 131:e35776. doi: 10.1002/cncr.35776 40019322 PMC11869935

[13] Scheepers E de Rooij BH Pijnenborg J van Huis-Tanja LH Ezendam N Hamaker ME . Perceived social support in patients with endometrial or ovarian cancer: a secondary analysis from the ROGY care study. Gynecologic Oncol. (2021) 160:811–6. doi: 10.1016/j.ygyno.2020.12.031 33454131

[14] McEwen BS Stellar E . Stress and the individual. Mechanisms leading to disease. Arch Intern Med. (1993) 153:2093–101. doi: 10.1016/b978-0-12-398270-4.00034-3 8379800

[15] Carbone JT Clift J Alexander N . Measuring allostatic load: approaches and limitations to algorithm creation. J Psychosomatic Res. (2022) 163:111050. doi: 10.1016/j.jpsychores.2022.111050 36228435

[16] Edes AN Edwards KL Wolfe BA Brown JL Crews DE . Allostatic load indices with cholesterol and triglycerides predict disease and mortality risk in zoo-housed western lowland gorillas (Gorilla gorilla gorilla). biomark Insights. (2020) 15:89755495. doi: 10.1177/1177271920914585 32425494 PMC7218307

[17] Bobba-Alves N Sturm G Lin J Ware SA Karan KR Monzel AS . Cellular allostatic load is linked to increased energy expenditure and accelerated biological aging. Psychoneuroendocrinology. (2023) 155:106322. doi: 10.1016/j.psyneuen.2023.106322 37423094 PMC10528419

[18] Xu C Huo D Liu Y Zhu Q Zhao J Liang J . Allostatic load and kidney cancer incidence and mortality: a genetic susceptibility and proteomic mediation analysis. BMC Cancer. (2025) 25:1575. doi: 10.1186/s12885-025-14980-6 41088055 PMC12523015

[19] McEwen BS . Stress, adaptation, and disease. Allostasis and allostatic load. Ann N Y Acad Sci. (1998) 840:33–44. doi: 10.1111/j.1749-6632.1998.tb09546.x 9629234

[20] Guidi J Lucente M Sonino N Fava GA . Allostatic load and its impact on health: a systematic review. Psychother Psychosomatics. (2021) 90:11–27. doi: 10.1159/000510696 32799204

[21] Wang F Skiba MB Follis S Liu N Bidulescu A Mitra AK . Allostatic load and risk of invasive breast cancer among postmenopausal women in the U.S [Journal Article. Prev Med. (2024) 178:107817. doi: 10.1016/j.ypmed.2023.107817 38097139

[22] Stabellini N Cullen J Bittencourt MS Moore JX Sutton A Nain P . Allostatic load/chronic stress and cardiovascular outcomes in patients diagnosed with breast, lung, or colorectal cancer [Journal Article. J Am Heart Assoc. (2024) 13:e33295. doi: 10.1161/JAHA.123.033295 38979791 PMC11292743

[23] Zhao J Xue E Zhou S Zhang M Sun J Tan Y . Allostatic load, genetic susceptibility, incidence risk, and all-cause mortality of colorectal cancer [Journal Article. Jnci-Journal Natl Cancer Institute. (2025) 117:134–43. doi: 10.1093/jnci/djae223 39271163

[24] Stabellini N Cullen J Bittencourt MS Moore JX Cao L Weintraub NL . Allostatic load and cardiovascular outcomes in males with prostate cancer [Journal Article; Research Support, N.I.H. Extramural; Research Support, Non-U.S. Gov't. Jnci Cancer Spectr. (2023) 7:pkad005. doi: 10.1093/jncics/pkad005 36752520 PMC10005613

[25] Li C Andrzejak SE Jones SR Williams BM Moore JX . Investigating the association between educational attainment and allostatic load with risk of cancer mortality among African American women. BMC Womens Health. (2023) 23:448. doi: 10.1186/s12905-023-02529-3 37620873 PMC10463695

[26] Obeng-Gyasi B Gokun Y Elsaid MI Chen JC Andersen BL Carson WE . The association between allostatic load and lymphedema in breast cancer survivors. Supportive Care Cancer. (2025) 33:311. doi: 10.1007/s00520-025-09362-4 40116971 PMC11928421

[27] Obeng-Gyasi S Elsaid MI Lu Y Chen JC Carson WE Ballinger TJ . Association of allostatic load with all-cause mortality in patients with breast cancer. JAMA Netw Open. (2023) 6:e2313989. doi: 10.1001/jamanetworkopen.2023.13989 37200034 PMC10196875

[28] Hoffmann JP Liu JA Seddu K Klein SL . Sex hormone signaling and regulation of immune function. Immunity. (2023) 56:2472–91. doi: 10.1016/j.immuni.2023.10.008 37967530

[29] Klusmann H Luecking N Engel S Blecker MK Knaevelsrud C Schumacher S . Menstrual cycle-related changes in HPA axis reactivity to acute psychosocial and physiological stressors - a systematic review and meta-analysis of longitudinal studies. Neurosci Biobehav Rev. (2023) 150:105212. doi: 10.1016/j.neubiorev.2023.105212 37149074

[30] Nunez SG Rabelo SP Subotic N Caruso JW Knezevic NN . Chronic stress and autoimmunity: the role of HPA axis and cortisol dysregulation. Int J Mol Sci. (2025) 26:9994. doi: 10.3390/ijms26209994 41155288 PMC12563903

[31] Hodes GE Bangasser D Sotiropoulos I Kokras N Dalla C . Sex differences in stress response: classical mechanisms and beyond. Curr Neuropharmacol. (2024) 22:475–94. doi: 10.2174/1570159X22666231005090134 37855285 PMC10845083

[32] Wang H Li C Chen L Zhang M Ren T Zhang S . Causal relationship between female reproductive factors, sex hormones and uterine leiomyoma: a Mendelian randomization study. Reprod Biomedicine Online. (2024) 48:103584. doi: 10.1016/j.rbmo.2023.103584 38061975

[33] Page MJ McKenzie JE Bossuyt PM Boutron I Hoffmann TC Mulrow CD . The PRISMA 2020 statement: an updated guideline for reporting systematic reviews. BMJ (Clinical Res Ed). (2021) 372:n71. doi: 10.1136/bmj.n71 33782057 PMC8005924

[34] Sterne JAC Savovi J Page MJ Elbers RG Blencowe NS Boutron I . RoB 2: a revised tool for assessing risk of bias in randomised trials. BMJ (Clinical Res Ed). (2019) 366:l4898. doi: 10.1136/bmj.l4898 31462531

[35] Borho L Bao R Elishaev E Dinkins KD O'Brien EE Berger J . Association of allostatic load with overall survival in epithelial ovarian cancer. Gynecologic Oncol. (2024) 186:204–10. doi: 10.1016/j.ygyno.2024.05.031 38843663 PMC11216875

[36] Chen JC Elsaid MI Handley D Anderson L Andersen BL Carson WE . Allostatic load as a predictor of postoperative complications in patients with breast cancer. NPJ Breast Cancer. (2024) 10:44. doi: 10.1038/s41523-024-00654-2 38866818 PMC11169387

[37] Chen JC Elsaid MI Handley D Plascak JJ Andersen BL Carson WE . Association between neighborhood opportunity, allostatic load, and all-cause mortality in patients with breast cancer. J Clin Oncol. (2024) 42:1788–98. doi: 10.1200/JCO.23.00907 38364197 PMC11095867

[38] Chen JC Handley D Elsaid MI Plascak JJ Andersen BL Carson WE . The implications of racialized economic segregation and allostatic load on mortality in patients with breast cancer. Ann Surg Oncol. (2024) 31:365–75. doi: 10.1245/s10434-023-14431-1 37865937

[39] Guan Y Anderson RT Gururaj S Cohn WF Chow PI Fuemmeler BF . Allostatic load and racial and rural disparities in breast cancer survival. JAMA Netw Open. (2025) 8:e2528019. doi: 10.1001/jamanetworkopen.2025.28019 40839266 PMC12371513

[40] Guan Y Shen J Lu J Fuemmeler BF Shock LS Zhao H . Association between allostatic load and breast cancer risk: a cohort study. Breast Cancer Research: Bcr. (2023) 25:155. doi: 10.1186/s13058-023-01754-w 38115125 PMC10729373

[41] Parente V Hale L Palermo T . Association between breast cancer and allostatic load by race: National Health and Nutrition Examination Survey 1999-2008. Psycho-Oncology. (2013) 22:621–8. doi: 10.1002/pon.3044 22290849

[42] Sangaramoorthy M Samayoa C Inamdar PP Roh JM Valice E Hong C . Association between neighborhood stressors and allostatic load in breast cancer survivors: the Pathways Study. Am J Epidemiol. (2025) 194:1264–74. doi: 10.1093/aje/kwae134 38896063 PMC12055468

[43] Xing CY Doose M Qin B Lin Y Carson TL Plascak JJ . Pre-diagnostic allostatic load and health-related quality of life in a cohort of Black breast cancer survivors. Breast Cancer Res Treat. (2020) 184:901–14. doi: 10.1007/s10549-020-05901-1 32914357 PMC7657984

[44] Xing CY Doose M Qin B Lin Y Plascak JJ Omene C . Prediagnostic allostatic load as a predictor of poorly differentiated and larger sized breast cancers among Black women in the Women's Circle of Health Follow-Up Study. Cancer Epidemiology Biomarkers Prevention: A Publ Am Assoc For Cancer Research Cosponsored by Am Soc Prev Oncol. (2020) 29:216–24. doi: 10.1158/1055-9965.EPI-19-0712 31719063 PMC6954339

[45] Zhao H Song R Ye Y Chow W Shen J . Allostatic score and its associations with demographics, healthy behaviors, tumor characteristics, and mitochondrial DNA among breast cancer patients. Breast Cancer Res Treat. (2021) 187:587–96. doi: 10.1007/s10549-021-06102-0 33507481 PMC12952051

[46] Bey GS Bowen MB Wu S Boykin M Bernard L Zhang Q . Allostatic load in endometrial cancer disparities. medRxiv. (2026) 2026.06.06.26355062. doi: 10.64898/2026.06.06.2635506231217447

[47] Fengfu C Hua X Chen X Liu M Chen A Wan B . Association between allostatic load and risk of breast carcinoma in situ in the UK biobank. Cancer causes control CCC. (2026) 37:102. doi: 10.1007/s10552-026-02186-5 42201432

[48] Sinha N Miller V Diggs A Broaddus E Bae-Jump V Lara OD . Impact of allostatic load on overall survival in patients with endometrial cancer. Cancer Med. (2026) 15:e71993. doi: 10.1002/cam4.71993 42210577 PMC13240357

[49] Bey GS Bowen MB Wu S Boykin M Bernard L Zhang Q . Allostatic load in endometrial cancer disparities. medRxiv preprint server Health Sci. (2026), 2026.06.06.26355062. doi: 10.64898/2026.06.06.26355062 42326808 PMC13278288

[50] Li C Howard SP Rogers CR Andrzejak S Gilbert KL Watts KJ . Allostatic load, educational attainment, and risk of cancer mortality among US men. JAMA Netw Open. (2024) 7:e2449855. doi: 10.1001/jamanetworkopen.2024.49855 39656456 PMC11632542

[51] Yang D Wheeler M Karanth SD Aduse-Poku L Leeuwenburgh C Anton S . Allostatic load and risk of all-cause, cancer-specific, and cardiovascular mortality in older cancer survivors: an analysis of the National Health and Nutrition Examination Survey 1999-2010. Aging Cancer. (2023) 4:74–84. doi: 10.1002/aac2.12064 37576467 PMC10421616

[52] Yuan D Wang M Bu S Mu T Li Y . Associations of socioeconomic factors and unhealthy lifestyles with allostatic load: a meta-analysis. Int J Behav Med. (2024) 31:772–86. doi: 10.1007/s12529-023-10235-5 37889389

[53] Cobbs CT Chesnut GT Shafi AA . Understanding racial disparities in prostate cancer: a multifaceted approach. Cancer Med. (2025) 14:e70979. doi: 10.1002/cam4.70979 40444484 PMC12123386

[54] Forster M Zhao F Bell S Mayer IA Arteaga CL Symmans WF . Impact of race, socioeconomic status, and clinicopathologic features on clinical outcomes in triple-negative breast cancer in the ECOG-ACRIN EA1131 trial. JCO Oncol Adv. (2026) 3:e2500177. doi: 10.1200/OA-25-00177 42145872 PMC13175121

[55] Harris AR Wang T Heng YJ Baker GM Le PA Wang J . Association of early menarche with breast tumor molecular features and recurrence. Breast Cancer research: BCR. (2024) 26:102. doi: 10.1186/s13058-024-01839-0 38886818 PMC11181557

[56] Bian Z Zhang R Yuan S Fan R Wang L Larsson SC . Healthy lifestyle and cancer survival: a multinational cohort study. Int J Cancer. (2024) 154:1709–18. doi: 10.1002/ijc.34846 38230569

[57] Park S Lee Y . Lifestyle factors affecting the prognosis of gynecological cancer: systematic review and meta-analysis. Cancer Causes Control: Ccc. (2025) 36:1633–45. doi: 10.1007/s10552-025-02059-3 40859088

[58] Khalil M Woldesenbet S Rashid Z Altaf A Zindani S Huang E . The hidden burden: impact of allostatic load on colorectal cancer surgery outcomes. Ann Surg Oncol. (2025) 32:7723–30. doi: 10.1245/s10434-025-17711-0 40544205 PMC12454506

[59] Barrett M Wilcox NS Huang A Levy R Demissei B Narayan V . Bearing allostatic load: insights into a more equitable future within cardio-oncology. Trends Mol Med. (2022) 28:1040–9. doi: 10.1016/j.molmed.2022.09.006 36207229

[60] Chiefari E Mirabelli M La Vignera S Tanyola S Foti DP Aversa A . Insulin resistance and cancer: in search for a causal link. Int J Mol Sci. (2021) 22:11137. doi: 10.3390/ijms222011137 34681797 PMC8540232

[61] Ge M Zhang W Zhang W Zhang Z Zeng X Deng L . Depression remodels tumor microenvironment to drive tumor progression: bio-behavioural signalling pathways and clinical interventions. J Adv Res. (2025) 86:S1232–2090. doi: 10.1016/j.jare.2025.11.001 41223987 PMC13453663

[62] Pu T Sun J Ren G Li H . Neuro-immune crosstalk in cancer: mechanisms and therapeutic implications. Signal Transduction Targeted Ther. (2025) 10:176. doi: 10.1038/s41392-025-02241-8 40456735 PMC12130251

[63] Karim AM Eun Kwon J Ali T Jang J Ullah I Lee Y . Triple-negative breast cancer: epidemiology, molecular mechanisms, and modern vaccine-based treatment strategies. Biochem Pharmacol. (2023) 212:115545. doi: 10.1016/j.bcp.2023.115545 37044296

[64] Vidović V Davidov I Ružić Z Erdeljan M Galfi Vukomanović A Blagojević B . Androgen receptors in human breast cancer and female canine mammary tumors. Molecules. (2025) 30:1411. doi: 10.3390/molecules30071411 40286049 PMC11990102

