# Supplementary material for: Allostatic load in female cancer patients: a systematic review
Source: Front Endocrinol (Lausanne). 2026 Sep 9;17:1881050. doi: 10.3389/fendo.2026.1881050 (PMC13597366; doi:10.3389/fendo.2026.1881050)
Supplement: Supplementary file 1 [file Table1.docx]

**Supplementary Table S1. Full search strategies for all databases**

| **Database** | **Search Date** | **Full Search String** | **Records Retrieved** |
| --- | --- | --- | --- |
| **PubMed** | June 30, 2025 | ("Neoplasms"[MeSH] OR cancer[Title/Abstract] OR tumor[Title/Abstract] OR tumour[Title/Abstract] OR malignancy[Title/Abstract] OR carcinoma[Title/Abstract]) AND ("Ovarian Neoplasms"[MeSH] OR "Uterine Cervical Neoplasms"[MeSH] OR "Endometrial Neoplasms"[MeSH] OR "Breast Neoplasms"[MeSH] OR ovarian[Title/Abstract] OR cervical[Title/Abstract] OR endometrial[Title/Abstract] OR breast[Title/Abstract] OR female[Title/Abstract] OR women[Title/Abstract] OR woman[Title/Abstract]) AND ("Allostatic Load"[MeSH] OR "allostatic load"[Title/Abstract] OR "allostatic overload"[Title/Abstract] OR "cumulative biological risk"[Title/Abstract] OR "biological wear and tear"[Title/Abstract]) | 46 |
| **Web of Science** | June 30, 2025 | TS=(("allostatic load" OR "allostatic overload" OR "cumulative biological risk" OR "biological wear and tear") AND (ovarian OR cervical OR endometrial OR breast OR female OR women OR woman) AND (cancer OR tumor OR tumour OR malignancy OR carcinoma)) | 127 |
| **Springer Link** | June 30, 2025 | ("allostatic load" OR "allostatic overload" OR "cumulative biological risk" OR "biological wear and tear")" and "(ovarian OR cervical OR endometrial OR breast OR female OR women OR woman)" and "(cancer OR tumor OR tumour OR malignancy OR carcinoma)" | 57 |
| **Wiley Online Library** | June 30, 2025 | ("allostatic load" OR "allostatic overload" OR "cumulative biological risk" OR "biological wear and tear")" in Title and "(ovarian OR cervical OR endometrial OR breast OR female OR women OR woman)" in Title and "(cancer OR tumor OR tumour OR malignancy OR carcinoma)" in Title | 2 |

**Supplementary Table S2. Quality Assessment of Included Studies Using the National Institutes of Health (NIH) Quality Assessment Tool for Observational Cohort and Cross-Sectional Studies**

| Criteria | Parente et al., 2013 | Xing et al., 2020a | Xing et al., 2020b | Zhao et al., 2021 | Guan et al., 2023 | Obeng-Gyasi et al., 2023 | Borho et al., 2024 | Chen et al., 2024a | Chen et al., 2024b | Chen et al., 2024c | F. Wang et al., 2024 | Guan et al., 2025 | Obeng-Gyasi et al., 2025 | Sangara  moorthy et al., 2025 | Bey et al., 2026 | Fengfu et al., 2026 | Sinha et al., 2026 |
| --- | --- | --- | --- | --- | --- | --- | --- | --- | --- | --- | --- | --- | --- | --- | --- | --- | --- |
| 1. Was the research question or objective in this paper clearly stated? | Yes | Yes | Yes | Yes | Yes | Yes | Yes | Yes | Yes | Yes | Yes | Yes | Yes | Yes | Yes | Yes | Yes |
| 2. Was the study population clearly specified and defined? | Yes | Yes | Yes | Yes | Yes | Yes | Yes | Yes | Yes | Yes | Yes | Yes | Yes | Yes | Yes | Yes | Yes |
| 3. Was the participation rate of eligible persons at least 50%? | CD | CD | CD | CD | CD | CD | CD | CD | CD | CD | Yes | CD | CD | CD | CD | CD | CD |
| 4. Were all the subjects selected or recruited from the same or similar populations (including the same time period)? Were inclusion and exclusion criteria for being in the study prespecified and applied uniformly to all participants? | Yes | Yes | Yes | Yes | Yes | Yes | Yes | Yes | CD | Yes | Yes | Yes | Yes | Yes | Yes | Yes | Yes |
| 5. Was a sample size justification, power description, or variance and effect estimates provided? | No | No | No | No | No | No | No | No | No | No | No | No | No | No | No | No | No |
| 6. For the analyses in this paper, were the exposure(s) of interest measured prior to the outcome(s) being measured? | Yes | Yes | Yes | No | Yes | Yes | Yes | Yes | No | Yes | Yes | Yes | Yes | NA | CD | Yes | CD |
| 7. Was the timeframe sufficient so that one could reasonably expect to see an association between exposure and outcome if it existed? | NA | Yes | Yes | NA | Yes | Yes | Yes | Yes | Yes | Yes | Yes | Yes | Yes | NA | Yes | Yes | Yes |
| 8. For exposures that can vary in amount or level, did the study examine different levels of the exposure as related to the outcome (e.g., categories of exposure, or exposure measured as continuous variable)? | Yes | Yes | Yes | Yes | Yes | Yes | Yes | Yes | Yes | Yes | Yes | Yes | Yes | Yes | Yes | Yes | Yes |
| 9. Were the exposure measures (independent variables) clearly defined, valid, reliable, and implemented consistently across all study participants? | Yes | Yes | Yes | Yes | Yes | Yes | Yes | Yes | Yes | Yes | Yes | Yes | Yes | Yes | Yes | Yes | Yes |
| 10. Was the exposure(s) assessed more than once over time? | No | No | No | No | No | No | No | No | No | No | No | No | No | Yes | No | No | No |
| 11. Were the outcome measures (dependent variables) clearly defined, valid, reliable, and implemented consistently across all study participants? | Yes | Yes | Yes | Yes | Yes | Yes | Yes | Yes | Yes | Yes | Yes | Yes | Yes | Yes | Yes | Yes | Yes |
| 12. Were the outcome assessors blinded to the exposure status of participants? | No | No | No | No | CD | No | No | No | No | No | No | No | No | No | No | No | No |
| 13. Was loss to follow-up after baseline 20% or less? | NA | CD | CD | NA | Yes | CD | CD | CD | CD | CD | Yes | CD | CD | NA | CD | Yes | CD |
| 14. Were key potential confounding variables measured and adjusted statistically for their impact on the relationship between exposure(s) and outcome(s)? | Yes | Yes | Yes | Yes | Yes | Yes | Yes | Yes | Yes | Yes | Yes | Yes | Yes | Yes | Yes | Yes | Yes |
| Quality Rating | Fair | Fair | Fair | Fair | Good | Fair | Fair | Fair | Fair | Fair | Good | Fair | Fair | Fair | Fair | Good | Fair |
| Additional Comments (If Poor, please state why): |  |  |  |  |  |  |  |  |  |  |  |  |  |  |  |  |  |

CD: Cannot determined; NA: Not applicable
